# Durvalumab Is Associated with Prolonged Progression-Free Survival, While Concomitant Chemoradiotherapy May Improve Both Locoregional and Local Control in Elderly Patients with Unresectable NSCLC Stage III: Subanalysis of the Austrian Radio-Oncological Lung Cancer Study Association Registry (ALLSTAR)

**DOI:** 10.3390/medsci13040305

**Published:** 2025-12-05

**Authors:** Franz Zehentmayr, Josef Karner, Markus Stana, Elvis Ruznic, Barbara Zellinger, Marisa Klebermass, Ayurzana Purevdorj, Georg Gruber, Danijela Minasch, Martin Heilmann, Raphaela Moosbrugger, Falk Roeder, Brane Grambozov

**Affiliations:** 1 Department of Radiation Oncology, Paracelsus Medical University, SALK, Müllner Hauptstraße 48, 5020 Salzburg, Austria; f.zehentmayr@salk.at (F.Z.); j.karner@salk.at (J.K.); m.stana@salk.at (M.S.); e.ruznic@salk.at (E.R.); f.roeder@salk.at (F.R.); 2 Institute of Pathology, Paracelsus Medical University, SALK, Müllner Hauptstraße 48, 5020 Salzburg, Austria; b.zellinger@salk.at; 3 Department of Radiation Oncology, Klinikum Ottakring, Montleartstraße 37, 1160 Vienna, Austria; marisa.klebermass@gesundheitsverbund.at; 4 Department of Radiation Oncology, Klinikum Hietzing-Rosenhügel, Wolkersbergenstraße 1, 1130 Vienna, Austria; ayurzana.purevdorj@gesundheitsverbund.at; 5 Department of Radiation Oncology, Ordensklinikum Linz, Seilerstätte 4, 4020 Linz, Austria; georg.gruber@ordensklinikum.at; 6 Department of Radiation Oncology, Comprehensive Cancer Centre, Medical University Innsbruck, Anichstraße 35, 6020 Innsbruck, Austria; danijela.minasch@tirol-kliniken.at; 7 Department of Radiation Oncology, Comprehensive Cancer Centre, Medical University Vienna, Währinger Gürtel 18-20, 1090 Vienna, Austria; martin.heilmann@akhwien.at; 8 Department of Pneumology, Paracelsus Medical University, SALK, Müllner Hauptstraße 48, 5020 Salzburg, Austria; r.moosbrugger@salk.at; 9 RadART—Institute for Research and Development on Advanced Radiation Technologies, Paracelsus Medical University, Müllner Hauptstraße 48, 5020 Salzburg, Austria

**Keywords:** radiation oncology, NSCLC, elderly, sequential radiotherapy, durvalumab

## Abstract

Introduction: The incidence of NSCLC increases with age, with a median of approximately 70 years at diagnosis. Historically, treatment strategies for locally advanced cancers have been developed predominantly in younger populations, often excluding elderly patients who may present with multiple comorbidities, severely impaired lung function, or decreased performance status, leading to a lack of age-relevant clinical data. Therefore, we performed a subanalysis of real-world data from the ALLSTAR study to investigate the impact of durvalumab and the radiation regimen (sequential versus concurrent) on clinical outcome in elderly patients with unresectable stage III NSCLC. Methods: We included a total of 171 patients in this subanalysis. All patients were diagnosed with unresectable stage III NSCLC. Patients were divided into two age groups, ≥70 (41%) and <70 years (59%). All of them received curative chemoradiotherapy with (66%) or without (34%) durvalumab. Results: Patients were followed up for a median time of 25.1 months (range: 3.3–52.1). In the elderly group, patients who did not receive durvalumab consolidation had a median PFS of 17 months (95%-CI: 12.4—not reached) and a higher risk of progression (HR = 2.2; 95%-CI: 1–4.6) than those treated with durvalumab, which had a median PFS of 37 months (95%-CI: 24.5—not reached). This difference was statistically significant (log rank *p* = 0.026). Moreover, the Cox model yielded a hazard ratio suggesting a higher risk of locoregional (HR = 3.8; 95%-CI: 1.28–11.48; log rank *p*-value =0.01) as well as local recurrence (HR = 5.5: 95%-CI: 1.67–18.1: *p*-value =0.002) in patients who received sequential chemoradiotherapy compared to those with concomitant chemoradiotherapy in the same age group. In an exploratory analysis based on a Mann–Whitney U test, we did not find significant difference in toxicity between the two age groups. Conclusions: Durvalumab was associated with prolonged progression-free survival, while concomitant radiotherapy showed a trend towards improvement in locoregional and local control in patients aged ≥70. There was no significant difference in treatment toxicity found in the exploratory Mann–Whitney U analysis between the two age groups.

## 1. Introduction

Worldwide, lung cancer remains the leading cause of cancer-related mortality [1]. Around 80% of cases are non-small-cell lung cancer (NSCLC), with a significant proportion being diagnosed at an advanced stage [1]. The incidence of NSCLC increases in the elderly with a median of approximately 70 years at diagnosis [2,3]. Historically, treatment strategies for advanced cancers have been developed predominantly in younger patient populations with select performance status, often excluding elderly patients who may present with multiple comorbidities, severely impaired lung function, or decreased performance status. This has limited age-relevant clinical data, especially from a radio-oncological perspective [4,5,6,7]. There are also physiological changes that accompany aging such as immune senescence [8] and altered drug metabolism [9], which pose additional challenges in treatment management. Primarily, but not exclusively, for these clinical reasons, over half of the patients in the ALLSTAR (Austrian Radio-Oncological Lung Cancer Study Association Registry) study, a real-world equivalent to the PACIFIC trial [4] within Austria, received sequential chemoradiotherapy [10,11]. In the original milestone study published by Antonia et al. [6], which established the standard of care for unresectable stage III NSCLC, patients were divided in age groups under or over 65 years, primarily based on clinical practice and statistical distribution. Only patients with good performance status (Eastern Cooperative Oncology Group: 0–1) were included, and the only primary clinical endpoints analyzed in regard to age were progression-free survival [4,6] and overall survival [6]. This was supplemented in a subanalysis [5], in which the age cutoff (70 years) was adjusted to the more commonly used geriatric definition of elderly patients [3,12,13,14] and the clinical endpoints were also expanded, but still did not include local and locoregional control. The latter two are decisive for evaluation of the efficacy of the radiation treatment. In addition, only patients who received concomitant chemoradiation were included, which is the gold standard but as previously mentioned does not fully reflect daily clinical practice [10,15]. Therefore, it is critical to investigate whether elderly patients have similar benefits from modern oncologic therapy regimens as their younger counterparts in a real-world clinical setting. To that end, we performed a subanalysis of real-world data from the ALLSTAR prospective registry [10] to investigate the impact of durvalumab as well as the chemoradiation regimen (sequential versus concurrent) on the clinical outcomes of patients aged ≥70 and <70 years with unresectable stage III NSCLC who were treated with chemoradiotherapy with or without durvalumab.

## 2. Methods

### 2.1. Patients and Design

This is a subanalysis of the Austrian Radio-Oncological Lung Cancer Study Association Registry (ALLSTAR), which is a multicenter, prospective registry collecting real-world data on the treatment of unresectable stage III NSCLC in Austria. The data was collected from March 2020 to April 2023. The Ethics Committee of the federal state of Salzburg approved ALLSTAR on 2nd March 2020 (Nr.1002/2019). Details on the design of the registry have already been described elsewhere [10]. For the current analysis, we included a total of 171 patients. Patients were divided into two age groups, ≥70 (41%) and <70 years (59%). Written informed consent was provided by all patients. All of them were at least 18 years old and had unresectable and histologically or cytologically verified NSCLC classified as UICC stage III (TNM version 8), which was treated with curative intent. Patients were included regardless of performance score. This subanalysis included only patients who received chemoradiation alone or followed by durvalumab, and those who received chemoradiation only. The diagnostic work-up consisted of whole-body CT scan and/or 18 F-FGD-PET-CT, cranial MRI, bronchoscopy, or transthoracic needle aspiration with endobronchial ultrasound for mediastinal lymph node staging and pulmonary function tests (PFTs). All of the clinical centers involved performed follow-ups, including clinical check-ups, contrast-enhanced thoracic CT scans, and PFTs. These were performed three months after the end of radiation treatment and every six months thereafter. Treatment decisions were made by consensus in the local tumor boards, which consisted of radiation oncologists, pulmonologists, medical oncologists, thoracic surgeons, radiologists, and pathologists. At each center, a local investigator certified by the Austrian board of radiation oncologists entered the data into the web-based data capture system. Toxicity was assessed using Common Terminology Criteria for Adverse Events version 5.0 and the follow-up visits were scheduled at 6-month intervals.

### 2.2. Treatment

Radiation treatment was delivered according to the established protocols at each center with 3D conformal radiotherapy (RT) as the minimal technical requirement. Advanced methods such as intensity-modulated radiation therapy (IMRT) or volumetric arc therapy (VMAT) were preferred. The fractionation regimen was specific to each center, i.e., normofractionated (standard; 60–66 Gy EQD2) and hypofractionated or twice-daily treatments (>60 Gy EQD2) were allowed. To ensure uniformity in the subanalysis, all doses were recalculated in a biologically equivalent dose in 2 Gy fractions (EQD2). Chemotherapy (platinum doublet) was given either concurrently or prior to irradiation, based on histology as well as the specific practices of each center. Patients with a PD-L1 expression of 1% or greater were considered PD-L1 positive and were eligible for immunotherapy, with treatment decisions made by the local tumor board. Further treatment details in the registry have already been described elsewhere [10].

### 2.3. Endpoints and Statistical Analysis

For data handling and analysis, the programming language R along with the tidyverse package was applied. Univariate and multivariate Cox regression as well as the Kaplan–Meier method were performed using the survival package. The index date for time-to-event analyses (OS, PFS, LRC, and LC) was set at the day of pathological diagnosis. Patients were divided into two age groups (≥70 and <70 years) and comparisons for each clinical endpoint were reported. For univariate Cox regression, the hazard ratios together with the 95%-CIs and log-rank test were reported. For multivariate Cox regression HRs, 95%-CIs and Wald statistic were provided. The following covariates were included in the multivariate model: chemoradiation regimen, gender, Eastern Cooperative Oncology Group (ECOG) performance status, histology, UICC stage, EQD2 prescribed to the primary tumor, EQD2 prescribed to the lymph nodes, volume of the tumor, volume of the lymph nodes, durvalumab treatment, esophagitis, and pneumonitis. A *p*-value below the 5% threshold was considered as statistically significant and the Benjamini–Hochberg procedure was used to adjust the *p*-values for multiple testing. Schoenfeld residuals were used to test the proportional hazard assumption of the Cox regression. In case of a violation of this assumption, restricted mean survival time (RMST) was additionally reported.

## 3. Results

The whole cohort included 171 patients (Table 1). Patients were divided into two age groups, ≥70 (70 patients; 41%) and <70 years (101 patients; 59%). In the age group <70 years, 57 patients were male (56%) and 44 female (44%), while in the ≥70 age group 45 patients were male (64%) and 25 female (36%).

Although patients were included regardless of performance status, the majority of them indeed had a good performance status (EOCG 0–1; 94%). As for histology, 69/101 (68%) patients had non-squamous and 32/101 (32%) had squamous NSCLC in the <70 years age group, whereas in the ≥70 years age group 29/70 (41%) of the patients had non-squamous and 41/70 (59%) had squamous NSCLC. All of the patients received curative chemoradiotherapy (sequential 71%; concurrent 25%; unknown 4%) with (66%) or without (34%) durvalumab consolidation. Further details regarding patient characteristics and treatment-related parameters are shown in Table 1.

**Figure 1:**
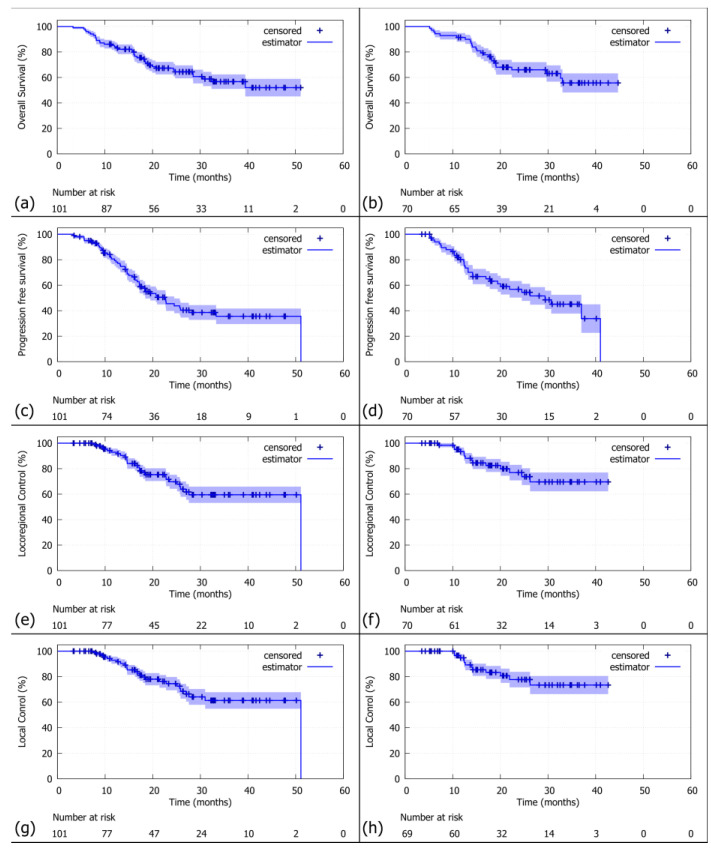
(**a**) the Kaplan–Meier curve for OS in the <70 years group, (**b**) the ≥70 years group. (**c**) The Kaplan–Meier curve for PFS in the <70 years group, (**d**) the ≥70 years group. (**e**) The Kaplan–Meier curve for LRC in the <70 years group, (**f**) in the ≥70 years group. (**g**) The Kaplan–Meier curve for LC in the <70 years group, (**h**) in the ≥70 years group.

In the <70 years group (Figure 1a), the median OS was not reached (95%-CI: 30.6—not reached) with a 2-year overall survival rate of 67% (95%-CI: 59–78) and a 3-year survival rate of 57% (95%-CI: 47–69). Similarly, the >70 years patient group (Figure 1b) did not reach the median survival (95%-CI: 32.6—not reached), with a 2-year survival rate of 66% (95%-CI: 56–79) and a 3-year survival rate of 56% (95%-CI: 43–72).

The median PFS was 22.7 months (95%-CI: 17.2–33.3) for the younger age group (Figure 1c) versus 29.4 months (95%-CI: 19.4—not reached) for the older age group (Figure 1d), respectively. The 2-year PFS was 46% (95%-CI: 36–58) for the younger and 57% (95%-CI: 45–71) for the older patient group, while the 3-year PFS was 36% (95%-CI: 26–50) in the younger and 45% (95%-CI: 33–62) in the older patient group.

The median LRC (Figure 1e,f) was 51.1 months (95%-CI: 27.6—not reached) in the younger and was not reached (95%-CI: not reached—not reached) in the older group. The 2-year LRC rate was 70% (95%-CI: 59–81) in the younger and 79% (95%-CI: 68–91) in the older patient group. At 3 years, LRC was 59% (95%-CI: 48–73) in the younger and 72% (95%-CI: 59–87) in the older patient group.

The median local control (Figure 1g,h) was 51.1 months (95%-CI: 31—not reached) for the younger patients and was not reached (95%-CI: not reached—not reached) for the older patients. The 2-year Kaplan–Meier estimate for LC was 74% (95%-CI: 65–85) in the younger and 79% (95%-CI: 69–92) in the older patient group. The 3-year LC rates were 61% (95%-CI: 50–75) in the younger and 75% (95%-CI: 62–90) in the older patient group.

**Figure 2:**
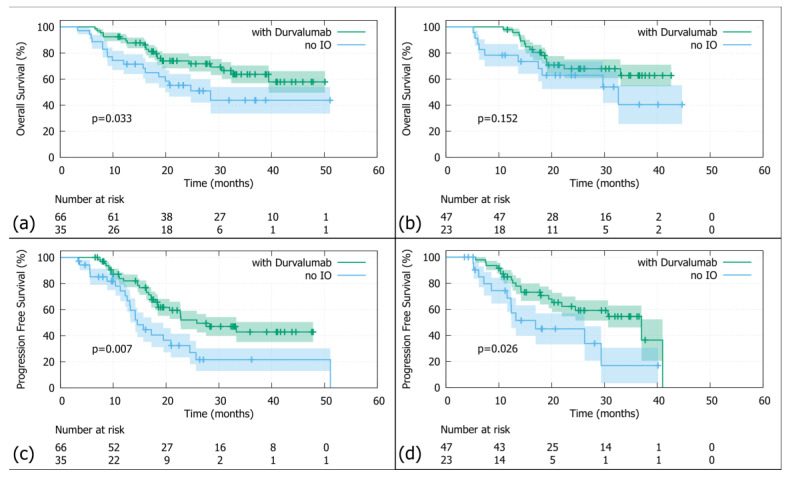
KM analysis of overall survival in patients aged <70 (**a**) and ≥70 years (**b**), as well as progression-free survival in patients aged <70 (**c**) and ≥70 years (**d**). KM = Kaplan–Meier; *p* = log-rank *p*-value; no IO = no immune checkpoint inhibitor (=no durvalumab).

In the <70s age group (Figure 2a), patients who did not receive durvalumab had a twofold higher risk of death compared to those who did (HR = 2; 95%-CI: 1–3.8). The median OS for patients without durvalumab was 28.5 months (95%-CI: 18.7—not reached), while those treated with durvalumab did not reach median survival (95%-CI: 39.5—not reached). This stratification was statistically significant (log-rank test: *p*-value = 0.03) and testing of the Schoenfeld residuals showed no violation of the proportional hazards assumption (*p*-value = 0.32). In contrast, in the ≥70 age group (Figure 2b), there was no significant difference in OS between those who received durvalumab and those who did not. The hazard ratio was 1.8 (95%-CI: 0.8–4), with a log-rank *p*-value of 0.152, indicating no statistical significance. In addition, the Schoenfeld residuals showed no violation of the proportional hazards assumption (*p*-value = 0.24). The median OS of patients treated with durvalumab was not reached (95%-CI: 33—not reached), while the median OS for those without durvalumab consolidation was 32.6 months (95%-CI: 18.2—not reached).

In the <70s age group (Figure 2c), patients who did not receive durvalumab had a median PFS of 16.3 months (95%-CI: 13.2–22.8) and a roughly twofold higher risk of progression (HR = 2.1; 95%-CI: 1.2–3.8) compared to those patients who received durvalumab (median PFS = 25.8 months; 95%-CI: 20.7—not reached). This difference was statistically significant (log rank *p* = 0.007) and testing of the Schoenfeld residuals indicated no significant violation of the proportional hazards assumption (*p*-value = 0.52). A similar trend was observed in the ≥70s age group (Figure 2d) where patients without durvalumab had a median PFS of 17 months (95%-CI: 12.4—not reached) and a roughly twofold higher risk of progression (HR = 2.2; 95%-CI: 1–4.6) than those treated with durvalumab, who had a PFS of 37 months (95%-CI: 24.5—not reached). This difference was also statistically significant (log rank *p* = 0.026) with Schoenfeld residuals again showing no significant violation of the proportional hazards assumption (*p*-value = 0.52).

**Figure 3:**
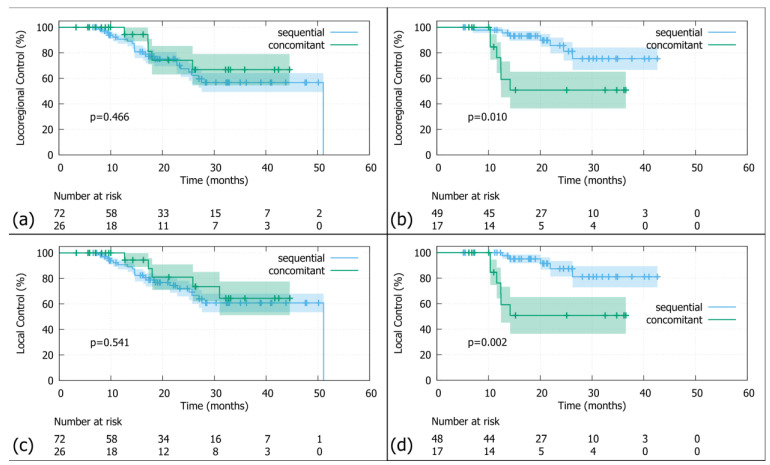
KM analysis of locoregional control in patients <70 (**a**) and ≥70 years (**b**), as well as local control in patients <70 (**c**) and ≥70 years of age (**d**). Although the Kaplan–Meier curve visually suggests better local and locoregional control for sequentially treated patients in the ≥70 group, this should be interpreted with caution as both the Cox analysis and RMST were unable to corroborate this finding. KM = Kaplan–Meier; *p* = log-rank *p*-value; no IO = no immune checkpoint inhibitor (=no durvalumab).

As shown in Figure 3a, the sequencing of radiotherapy in the <70s age group (median LRC = 51.1 months; 95%-CI: 27.6—not reached) did not significantly impact locoregional control (HR = 0.7; 95%-CI: 0.26–1.8; log rank *p*-value = 0.47). Testing of the Schoenfeld residuals confirms no violation of the proportional hazard assumption (*p*-value = 0.54). In the ≥70s group (median LRC not reached in either group; Figure 3b), the Cox model produced a hazard ratio suggesting a higher risk of locoregional recurrence in the sequential chemoradiotherapy group (median LC nor reached HR = 3.8; 95%-CI: 1.28–11.48; log rank *p*-value = 0.01), but the Kaplan–Meier curves visually appeared to show the opposite pattern. This discrepancy is probably due to violation of the proportional hazard assumption, as indicated by the Schoenfeld residuals (*p*-value = 0.054), making Cox analysis an unreliable summary measure for this specific dataset.

Further analysis using restricted mean survival time over the first 36 months revealed a significant difference; namely, patients in the sequential radiation treatment group had an RMST without locoregional recurrence of 24.1 months compared to 32.1 months in the concomitant treatment group. This difference of approximately 8 months was statistically significant (RMST difference: −7.98 months, 95% CI: −15.26 to −0.69, *p* = 0.03). Additionally, patients receiving sequential radiation treatment also had more restricted mean time lost because of locoregional recurrence (RMTL: 11.86 vs. 3.89 months, *p*-value = 0.01). These results suggest a reduction in locoregional control with the sequential regimen, thereby indicating a trend of advantage of concomitant chemoradiation treatment over sequential. This is consistent with the findings from the Cox analysis (hazard ratio) and RMST rather than the Kaplan–Meier curve.

The sequencing of radiotherapy did not have an impact on locoregional control in the <70s age group (Figure 3c; HR = 0.74, 95%-CI: 0.28–2; log-rank *p*-value = 0.54). Testing of the Schoenfeld residuals showed no significant violation of the proportional hazards assumption (*p*-value = 0.54). In this age group, patients who were treated with sequential chemoradiotherapy did not reach the median local control (95%-CI: 31—not reached) compared to the concomitant group, which had a median LC of 51.1 months (95%-CI: 27.6—not reached).

In contrast, in the ≥70s age group, patients who received sequential chemoradiotherapy (median LC not reached; 95%-CI: 12.4—not reached) had a significantly higher risk of local relapse with an HR of 5.5 (95%-CI: 1.67–18.1) compared to those with concomitant chemoradiotherapy (median LC not reached; 95%-CI: not reached—not reached; Figure 3d), although the Kaplan–Meier curves visually suggested otherwise. This discrepancy may once more be attributed to the violation of the proportional hazards assumption, as indicated by the Schoenfeld residuals *p*-value = 0.015), making Cox analysis an unreliable summary measure for this specific dataset as well. Analysis using restricted mean survival time over the first 36 months resulted in an RMST of 24.1 months compared to 33.1 months in the concomitant patient group. This difference of approximately 9 months (95%-CI: 16.2–1.8) was statistically significant (*p*-value = 0.014). In addition, patients receiving sequential radiation regimen experienced more restricted mean time lost due to local recurrence (RMTL 11.9 vs. 2.9, *p*-value = 0.005). These results indicate a trend of superiority of concomitant chemoradiation treatment over sequential. This aligns very well with the findings from the Cox proportional hazards regression analysis and RMST, rather than those suggested by the Kaplan–Meier curve.

The multivariate analyses including 12 variables showed that durvalumab significantly impacted OS in the <70s age group (HR: 3.1 95%-CI: 1.3–7.3; *p*-value < 0.01; adj. *p*-value < 0.054). Testing of the Schoenfeld residuals in the multivariate setting revealed no violation of the proportional hazards assumption (*p*-value = 0.285). In the ≥70s age group, patients who did not receive consolidation with durvalumab had a significantly shorter PFS than durvalumab patients (HR: 2.71 (95%-CI: 1.05–7); *p*-value = 0.04). Although the multivariate analysis showed a significant association, it did not remain significant after adjustment for multiple comparisons (adj. *p*-value = 0.25), indicating that the result should be interpreted with caution. Schoenfeld residuals testing revealed no violation of the proportional hazards assumption (*p*-value = 0.543) in the multivariate analysis.

While concomitant chemoradiotherapy improved both locoregional and local control in the ≥70s age group in the UVA, these effects did not persist in the multivariate Cox regression models (Table 2), indicating that the observed benefit was not independent of the other covariates included in the multivariate analysis. Other variables that also significantly impacted the specific clinical endpoints are shown in Table 2. Additionally, we have provided the hazard ratios and 95% confidence intervals for all variables in the Supplementary Table S1–S4.

In terms of toxicity, 49/101 (48.5%) patients in the <70 age group had grade 1–3 esophagitis and 37/101 (36%) had grade 1–4 pneumonitis. Among the patients with grade 1–3 esophagitis, 13 (12.9%) had grade 1, 33 had grade 2 (32.7%), and 3 (3%) had grade 3. Among the patients with grade 1–4 pneumonitis, 14 (13.9%) had grade 1, 21 had grade 2 (20.8%), 1 (1%) had grade 3, and 1 had grade 4 (1%) pneumonitis. In the ≥70 age group, 33/70 (47%) had grade 1–2 esophagitis and 25/70 (36%) had grade 1–3 pneumonitis. Among the patients with esophagitis, 7 (10%) had grade 1 and 26 had grade 2 (37.1%). Among the patients with pneumonitis, 7 (10%) had grade 1, 16 (22.9%) had grade 2, and 2 (2.9%) suffered from grade 3 pneumonitis. We did not observe any grade 5 toxicity event. An exploratory Mann–Whitney-U-Test showed no significant difference in toxicity (esophagitis *p* = 0.469; pneumonitis *p* = 0.364) between the two age groups (Table 3). This comparison does not incorporate time-to-event data and should therefore be interpreted cautiously and as exploratory.

## 4. Discussion

Our subanalysis shows that consolidation therapy with durvalumab was associated with improved prolonged PFS in elderly patients with unresectable stage III NSCLC, whereas a concomitant chemoradiotherapy regimen showed a trend of improvement in both local and locoregional control in the same population. These results provide valuable clinical insights, particularly from a radiation oncology perspective, in a patient cohort that represents almost half of the real-world patient population.

Although other cutoffs have been proposed, most oncological studies have considered an age of ≥70 years as the age at which frailty and treatment-related complications may increase and geriatric assessment becomes reasonable [14,16]. Accordingly, we used this practical age cutoff to define elderly patients in our study as well. The PACIFIC trial established durvalumab following concurrent chemoradiotherapy (cCRT) as the standard treatment in unresectable NSCLC stage III, showing significant improvements in both PFS and OS [4,6]. Both clinical endpoints were statistically significant in patients who received durvalumab in addition to chemoradiation over the placebo cohort (only chemoradiation) regardless of age. However, as groundbreaking as these findings were, elderly patients were not specifically evaluated beyond a basic age stratification at 65 years, making the extrapolation of the results to the elderly quite difficult. Nevertheless, a subsequent subanalysis [5] of the PACIFIC trial narrowed this gap, demonstrating the benefits of consolidation treatment with durvalumab in elderly patients (70 years or above). In the subanalysis by Socinski and colleagues [5], consolidation therapy with durvalumab significantly impacted PFS regardless of age, while OS in the ≥70 age group only showed a trend towards improvement, but was not significant (HR: 0.78; 95%-CI: 0.50–1.22). These results were consistent with our study in relation to both PFS and OS. This could be explained by several factors, of biological as well as statistical nature. Age-related immunosenescence has already been the topic of discussion in the thoracic oncology research community for some time. It is often described as a complex network of multicellular and metabolic interactions that lead to an age-related decline in immune cell competence, affect tumor microenvironment, and ultimately result in impaired response to immunotherapy [8]. This could be a possible explanation for our results regarding OS. However, the elderly cohort in our subanalysis was even smaller than that of Socinski et al. [5], the multivariate analysis was not able to demonstrate a significant overall survival benefit of durvalumab. Hence, we believe that this statistical insignificance might have been due to the small sample size of elderly patients and consequent loss of statistical power and/or deaths attributable to comorbidities or senility rather than immunosenescence.

With respect to toxicity, our real-world data showed no significant difference in toxicity between the two age groups. Studies have reported a numerically higher incidence of treatment-related toxicity events in older patients; however, neither study performed significance testing, so these results should be interpreted solely as descriptive trends [5,17]. The same trend was also observed in a meta-analysis by Wang et al. [18], but only for grade 1–2 toxicities. Furthermore, their results demonstrated that the rate of grade 3 or higher toxic events was not significantly different from the rate in younger patients [18], confirming our findings. Considering that elderly patients are often multimorbid with severely impaired lung function and reduced performance status, it would be reasonable to assume that they might be more prone to treatment-related toxicities than their younger counterparts [19,20]; however, our results suggest otherwise, once more underlining the importance of considering biological rather than chronological age in treatment decision-making.

While concomitant chemoradiotherapy followed by durvalumab is considered the gold standard [4,6,21], local control and locoregional control, which are decisive clinical endpoints for assessing efficacy of radiotherapy, were not analyzed for elderly patients in the existing milestone studies [4,5,6,15]. The tumor volume and/or location can pose significant challenges for radiation oncologists when planning radiation treatment, especially in elderly multimorbid patients with often compromised pulmonary reserves, since treatment-related toxicity can result in discontinuation of therapy. Another key factor is the limited radiotherapy and bed capacity in many hospitals, which makes concurrent treatment difficult to organize. In real-world settings, this often makes sequential radiotherapy a frequently used alternative. For that reason, we analyzed the different radiation therapy regimens (sequential versus concomitant) in relation to both clinical endpoints (local control and locoregional control) in this patient cohort. A meta-analysis performed by Auperin and colleagues [21] in the pre-immunotherapy era comprising 1205 patients with locally advanced NSCLC demonstrated that concomitant chemoradiotherapy significantly improved overall survival and locoregional control (progression-free survival showed only a trend towards improvement) in comparison to sequential chemoradiotherapy. This is in line with our observation in the immunotherapy era, which showed a trend toward improvement (significant in the UVA, but insignificant in MVA) of both local and locoregional control with concomitant chemoradiotherapy followed by durvalumab. Given that locoregional progression remains a dominant pattern of failure in stage III NSCLC [21] and elderly patients are often at risk of under-treatment [14], concomitant chemoradiotherapy may provide enhanced clinical benefit in this subgroup compared to the sequential treatment approach while maintaining a similar toxicity profile as shown in our study.

This study has several limitations, including its potential selection bias. Unlike other subanalyses [5], this subanalysis included patients regardless of performance status; however, over 90 percent had a good performance status. Data on quality of life as well as comprehensive geriatric assessment were not systematically collected, which is clinically important in this patient population.

Despite the prospective and multicenter design of this registry, unmeasured confounding factors may still exist due to variations in clinical decision-making, institutional protocols, and patient characteristics inherent to real-world settings. In addition, the potential for immortal time bias further limits the validity of the findings. In this context, for local and locoregional control, outcomes were analyzed without modeling the competing event of death, which given the 6-month follow up intervals may overestimate control. Moreover, in terms of toxicity, the exploratory analysis using the Mann–Whitney U test does not account for time-to-event data and should therefore be interpreted with caution. Consequently, the observed associations in this study should be considered exploratory rather than confirmatory and should not be interpreted as causal. In spite of these constraints, our results offer valuable real-world clinical insight into treatment paradigms in the elderly.

## 5. Conclusions

Durvalumab was associated with improved progression-free survival, while concomitant chemoradiotherapy showed a trend towards improvement in relation to locoregional and local control in elderly patients. There was no significant difference in treatment toxicity found in the exploratory Mann–Whitney U analysis between the two age groups. This treatment approach could potentially optimize both local and systemic control with acceptable treatment tolerability for this increasing patient subpopulation.

## Figures and Tables

**Table 1:** The patient characteristics and treatment-related parameters in the <70 years and ≥70 years age groups.

	Patient Characteristics and Treatment-Related Parameters			
			<70 (N = 101)	≥70 (N = 70)
Parameters	Gender	male	57 (56%)	45 (64%)
		female	44 (44%)	25 (36%)
	Histo	nonSCC	69 (68%)	29 (41%)
		SCC	32 (32%)	41 (59%)
	ECOG	0–1	95 (94%)	65 (93%)
		2–3	6 (6%)	5 (7%)
	CRT sequence	sCRT	72 (71%)	49 (70%)
		cCRT	26 (26%)	17 (25%)
		unknown	3 (3%)	4 (6%)
	UICC	IIIa + IIIb	59 (58%)	48 (69%)
		IIIc	42 (42%)	22 (31%)
	durvalumab	yes	66 (65%)	47 (67%)
		no	35 (35%)	23 (33%)
	EQD2 Tumor (Gy)	median	65.6	65
		range	24.8–100	32.5–100
	EQD2 LN (Gy)	median	57.3	57.3
		range	0–70	0–81.25
	Tumor GTV (ml)	median	51.8	49.7
		range	0–589.3	0.24–784.1
	LN GTV (ml)	median	25.2	31.75
		range	0–473	0–285

**Table 2:** (**a**) Multivariate and univariate analyses of variables in relation to OS, PFS, LRC, and LC in the < 70 years age group. (**b**) Multivariate and univariate analyses of variables in relation to OS, PFS, LRC, and LC in the ≥ 70 years age group. Abbreviations: n.s., not significant; adj., adjusted.

(a) Baseline Characteristics < 70 Years (n = 101)								
**Parameters**	OS		PFS		LRC		LC	
	UVA	MVA	UVA	MVA	UVA	MVA	UVA	MVA
Gender	n.s.	n.s.	n.s.	n.s.	n.s.	n.s.	n.s.	n.s
Histo	n.s.	n.s.	n.s.	n.s.	HR: 3.4 (95%-CI: 1.5–7.6); *p*-value < 0.003	HR: 1.2 (95%-CI: 1.4–8); *p*-value < 0.01; adj. *p*-value < 0.1	HR: 4.3 (95%-CI: 1.8–10.4); *p*-value < 0.001	HR: 4.3 (95%-CI: 1.7–11.2); *p*-value < 0.01; adj. *p*-value < 0.03
ECOG	HR 2 (95%-CI: 1.2–3.3); *p*-value < 0.004	n.s.	HR: 1.57 (95%-CI: 0.6–2.5) *p*-value = 0.05	n.s.	n.s.	n.s.	n.s.	n.s
CRT sequence	n.s.	HR 2.4; 95%-CI: 0.97–5.9; *p*-value < 0.06; adj. *p*-value < 0.23	n.s	n.s.	n.s.	n.s.	n.s.	n.s
UICC	n.s.	n.s.	HR: 2.2 (95%-CI: 1.18–4); *p*-value = 0.01	n.s	n.s.	n.s.	HR: 2.8 (95%-CI: 1.1–7.1); *p*-value = 0.02	HR: 3.3 (95%-CI: 1.06–10.1); *p*-value < 0.04; adj. *p*-value < 0.17
Durvalumab	HR: 2 (95%-CI: 1.04–3.9); *p*-value: 0.03	HR: 3.1 95%-CI: 1.3–7.3; *p*-value < 0.01; adj. *p*-value < 0.054	HR: 2.2 (95%-CI: 1.2–3.8); *p*-value = 0.007	n.s.	n.s.	n.s.	n.s.	n.s
EQD2 tumor (Gy)	HR: 1.04 (95%-CI: 1.01–1–07); *p*-value = 0.006	HR: 1.05; 95%-CI: 1.02–1.08; *p*-value < 0.001; adj. *p*-value < 0.01	n.s.	n.s.	n.s.	n.s.	n.s.	n.s
EQD2 LN (Gy)	n.s.	n.s.	n.s.	n.s.	n.s.	n.s.	n.s.	n.s
Tumor GTV (mL)	n.s.	n.s.	HR 1.003 (95%-CI: 1.001–1.005); *p*-value = 0.006	n.s.	n.s.	n.s.	n.s.	n.s
LN GTV (mL)	n.s.	n.s.	n.s.	n.s.	n.s.	n.s.	n.s.	n.s
Pneumonitis	HR: 0.6 (95%-CI: 0.37–0.98); *p*-value: 0.04	n.s.	n.s.					
Esophagitis	n.s.	n.s.	n.s.			HR: 0.6 (95%-CI: 0.38–0.99); *p*-value 0.046; adj. *p*-value = 0.27	HR: 0.65 (95%-CI: 0.4–1.03); *p*-value 0.06	HR: 0.59 (95%-CI: 0.35–0.98), *p*-value = 0.04; adj. *p*-value = 0.17
**(b) Baseline Characteristics ≥ 70 years (n = 70)**								
**Parameters**	OS		PFS		LRC		LC	
	UVA	MVA	UVA	MVA	UVA	MVA	UVA	MVA
Gender	n.s.	n.s.	n.s.	n.s.	n.s.	HR: 15.6 (95%-CI: 1.96–124.5); *p*-value = < 0.01; adj. *p*-value < 0.06	n.s.	HR: 9.1 (95%-CI: 1.4–60.7); *p*-value = 0.2; adj. *p*-value = 0.1;
Histo	n.s.	n.s.	n.s.	n.s.	n.s.	HR: 17.7 (95%-CI: 2.6–123); *p*-value < 0.01; adj. *p*-value = 0.044	n.s.	HR: 11.2 (95%-CI: 1.5–86); *p*-value < 0.02; adj. *p*-value = 0.11
ECOG	n.s.	n.s.	n.s.	n.s.	n.s.	n.s.	n.s.	n.s.
CRT sequence	n.s.	n.s.	n.s.	n.s.	HR: 3.8 (95%-CI: 1.28–11.5); *p*-value = 0.01	n.s.	HR: 5.5 (95%-CI: 1.7–18.1); *p*-value = 0.002	n.s.
UICC	n.s.	n.s.	n.s.	n.s.	n.s.	HR: 0.26 (95%-CI: 0.05–1.26); *p*-value < 0.1; adj. *p*-value < 0.19	n.s.	HR: 0.19 (95%-CI: 0.03–1.02; *p*-value = 0.05; adj. *p*-value = 0.11.
Durvalumab	n.s.	n.s.	HR: 2.1 (95%-CI: 1.01–4.5); *p*-value = 0.04	HR: 2.71 (95%-CI: 1.05–7); *p*-value = 0.04; adj. *p*-value = 0.25	n.s.	HR: 5.28 (95%-CI: 0.87–32.1); *p*-value 0.07; adj. *p*-value = 0.17	n.s.	n.s.
EQD2 tumor (Gy)	n.s.	n.s.	n.s.	HR: 0.9 (95%-CI: 0.89–1.003); *p*-value = 0.063; adj. *p*-value = 0.25	n.s.	n.s.	n.s.	n.s.
EQD2 LN (Gy)	n.s.	n.s.	n.s.	n.s.	n.s.	HR: 1.2 (95%-CI: 0.84–1.12); *p*-value = 0.034; adj. *p*-value = 0.1	HR: 1.09 (95%-CI: 1.002–1.19); *p*-value 0.07	HR: 1.2 (95%-CI: 1.007–1.4); *p*-value = 0.04; adj. *p*-value = 0.1
Tumor GTV (mL)	n.s.	n.s.	n.s.	HR: 0.994 (95%-CI: 0.99–1.000); *p*-value = 0.054; adj. *p*-value = 0.25	n.s.	n.s.	n.s.	n.s.
LN GTV (mL)	n.s.	n.s.	n.s.	n.s.	HR: 1.007 (95%-CI: 1.001–1.014); *p*-value 0.03	HR: 1.03 (95%-CI: 1.004–1.05); *p*-value < 0.02; adj. *p*-value < 0.08	HR: 1.009 (95%-CI: 1.002–1.016); *p*-value = 0.01	HR: 1.025 (95%-CI: 1.0014–1.05); *p*-value < 0.04; adj. *p*-value = 0.1
Pneumonitis	n.s.	n.s.	n.s.	n.s.	n.s.	n.s.	n.s.	HR: 3.4 (95%-CI: (1.02–11.2); *p*-value 0.046; adj. *p*-value < 0.11
Esophagitis	n.s.	n.s.	n.s.	n.s.	n.s.	n.s.	HR: 1.9 (95%-CI: 0.998–3.6); *p*-value = 0.04	

**Table 3:** The toxicity in both age groups.

Toxicity						
		<70 Years		≥ 70 Years		
		N = 101	%	N = 70	%	Mann–W-U, 2-sided
Esophagitis	Grade 1	13	12.9	7	10.0	*p* = 0.469
	Grade 2	33	32.7	26	37.1	
	Grade 3	3	3.0	0	0.0	
	Grade 4	0	0.0	0	0.0	
	Grade 5	0	0.0	0	0.0	
Pneumonitis	Grade 1	14	13.9	7	10.0	*p* = 0.364
	Grade 2	21	20.8	16	22.9	
	Grade 3	1	1.0	2	2.9	
	Grade 4	1	1.0	0	0.0	
	Grade 5	0	0.0	0	0.0	

## Data Availability

The data underlying this study are available from the corresponding author upon justified request. They are not publicly available due to privacy and ethical restrictions related to participant confidentiality.

## Supplementary Materials

The following supporting information can be downloaded at https://www.mdpi.com/article/10.3390/medsci13040305/s1, Table S1: Multivariate and univariate analyses of variables in relation to OS in the <70 years as well as ≥70 years age group.; Table S2: Multivariate and univariate analyses of variables in relation to PFS in the <70 years as well as ≥70 years age group. Table S3: Multivariate and univariate analyses of variables in relation to LRC in the <70 years as well as ≥70 years age group. Table S4: Multivariate and univariate analyses of variables in relation to LC in the <70 years as well as ≥70 years age group.

## Abbreviations

The following abbreviations are used in this manuscript:

ALLSTAR	Austrian Radio-Oncological Lung Cancer Study Association Registry
CRT	Chemoradiotherapy
cCRT	Concomitant chemoradiotherapy
EQD2	Biologically equivalent dose in 2 Gy fractions
GTV	Gross tumor volume
ICI	Immune-checkpoint inhibitor
IMRT	Intensity-modulated radiotherapy
LC	Local control
mOS	Median overall survival
MVA	Multivariate analysis
OAR	Organs at risk
OS	Overall survival
PD-L1	Programmed death ligand 1
PFS	Progression-free survival
PS	Performance score
RT	Radiotherapy
SCC	Squamous cell carcinoma
SoC	Standard of care
sCRT	Sequential chemoradiotherapy
VMAT	Volumetric arc therapy
