# Supplementary material for: Durvalumab Is Associated with Prolonged Progression-Free Survival, While Concomitant Chemoradiotherapy May Improve Both Locoregional and Local Control in Elderly Patients with Unresectable NSCLC Stage III: Subanalysis of the Austrian Radio-Oncological Lung Cancer Study Association Registry (ALLSTAR)
Source: Med Sci (Basel). 2025 Dec 5;13(4):305. doi: 10.3390/medsci13040305 (PMC12734908; doi:10.3390/medsci13040305)
Supplement: Supplementary file 1 [file medsci-13-00305-s001.zip › medsci-3938757-supplementary.pdf]

Supplementary tables

Supplementary Table S1. Multivariate and univariate analyses of variables in relation to OS in the < 70 years as well as ≥ 70 years age group. Abbreviations: adj., adjusted. BH., Benjamini-Hochberg and PH., proportional hazards. SCC., Squamous Cell Carcinoma. CRT., chemoradiotherapy. EQD2., Equivalent Dose in 2 Gray (Gy) fractions. LN., lymph nodes. GTV., gross tumor volume.

**Treatment characteristics**										
younger (< 70 years) patients (N = 95, events = 37)										
Factor (# of cases)	Univariate analysis					Multivariate analysis				
	P value (Log-rank)	HR	95% CI		P value (PH assumption)	P value (Wald) / BH adj.	HR	95% CI		P value (PH assumption)
			Lower	Upper				Lower	Upper	
Gender (female: 40; male: 55)	0.4	0.76	0.39	1.45	0.21	0.057/0.17	0.48	0.22	1.02	0.1
Histo (non SCC: 54; SCC: 41)	0.3	1.4	0.75	2.75	0.45	0.55/0.73	1.26	0.59	2.7	0.15
ECOG (min: 0; median: 0; max: 3)	0.007	1.96	1.19	3.2	0.77	0.16/0.24	1.5	0.85	2.75	0.36
CRT sequence (concomitant: 71; sequential: 24)	0.6	1.23	0.6	2.55	0.008	0.058/0.23	2.4	0.97	6	0.008
UICC stage (IIIa: 38; IIIb: 57)	0.2	1.5	0.75	3	0.24	0.16/0.24	1.8	0.79	4.1	0.07
Durvalumab (durvalumab: 63; no durvalumab: 32)	0.03	2	1.04	3.9	0.31	<0.01/0.05	3.1	1.3	7.3	0.27
Tumor EQD2 (min: 24.8; median: 66; mean: 64.93; max: 100)	0.006	1.04	1.013	1.069	0.24	<0.001/<0.01	1.1	1.02	1.08	0.21
LN EQD2 (min: 0; median: 57.29; mean: 50.98; max: 70)	0.3	1.011	0.99	1.032	0.89	0.78/0.85	0.996	0.968	1.025	0.997
Tumor GTV (min: 0.8; median: 48.42; mean: 76.79; max: 589.3)	0.7	1.001	0.997	1.004	0.19	0.96/0.96	1.0001	0.996	1.004	0.32
LN GTV (min: 0; median: 25.2; mean: 48.73; max: 473)	0.06	1.005	0.9999	1.01	0.047	0.15/0.24	1.0045	0.998	1.011	0.13
Pneumonitis (min: 0; median: 0; max: 4)	0.04	0.6	0.368	0.98	0.63	0.08/0.19	0.6	0.356	1.059	0.47
Esophagitis (min: 0; median: 0; max: 3)	0.5	1.12	0.796	1.54	0.31	0.62/0.75	1.094	0.76	1.57	0.59
elderly (≥ 70 years) patients (N = 65; events = 24)										
Factor (# of cases)	Univariate analysis					Multivariate analysis				
	P value (Log-rank)	HR	95% CI		P value (PH assumption)	P value (Wald) / BH adj.	HR	95% CI		P value (PH assumption)
			Lower	Upper				Lower	Upper	

Gender<br>(female: 24; male: 41)	0.7	1.18	0.5	2.7	0.044	0.65/0.98	1.2	0.48	3.22	0.07
Histo<br>(non SCC: 37; SCC: 28)	0.9	1.05	0.467	2.38	0.82	0.75/0.98	1.17	0.45	3	0.78
ECOG<br>(min: 0; median: 0; max: 2)	0.3	1.41	0.7	2.8	0.37	0.26/0.98	1.6	0.7	3.7	0.53
CRT sequence<br>(concomitant: 48; sequential: 17)	1	0.989	0.4	2.4	0.83	0.85/0.98	0.9	0.3	2.7	0.995
UICC stage<br>(IIIa: 19; IIIb: 46)	0.6	1.29	0.5	3.2	0.98	0.69/0.98	1.25	0.43	3.56	0.89
Durvalumab<br>(durvalumab: 45; no durvalumab: 20)	0.08	2	0.9	4.6	0.17	0.35/0.98	1.59	0.59	4.2	0.11
Tumor EQD2<br>(min: 32.5; median: 65; mean: 63.04; max 100)	0.1	0.97	0.94	1.007	0.23	0.21/0.98	0.96	0.9	1.02	0.21
LN EQD2<br>(min: 0; median: 57.29; mean 49.16; max: 70)	0.7	1.005	0.979	1.031	0.17	0.4/0.98	1.017	0.978	1.06	0.15
Tumor GTV<br>(min: 0.24; median: 49; mean: 92.6; max: 492.9)	0.5	1.001	0.998	1.004	0.61	0.9/0.98	0.9998	0.995	1.004	0.51
LN GTV<br>(min: 0; median: 30; mean: 58.89; max: 285)	0.5	1.002	0.996	1.007	0.99	0.98/0.98	1.0001	0.99	1.008	0.96
Pneumonitis<br>(min: 0; median: 0; max: 3)	0.8	1.06	0.697	1.625	0.16	0.64/0.98	1.11	0.71	1.72	0.13
Esophagitis<br>(min: 0; median: 0; max: 2)	0.3	1.28	0.83	1.97	0.26	0.4/0.98	1.2	0.73	2.08	0.25

Supplementary Table S2. Multivariate and univariate analyses of variables in relation to PFS in the < 70 years as well as ≥ 70 years age group. Abbreviations: adj., adjusted. BH., Benjamini-Hochberg and PH., proportional hazards. SCC., Squamous Cell Carcinoma. CRT., chemoradiotherapy. EQD2., Equivalent Dose in 2 Gray (Gy) fractions. LN., lymph nodes. GTV., gross tumor volume.

**Treatment characteristics**										
younger (< 70 years) patients (N = 95, events = 50)										
	Univariate analysis					Multivariate analysis				
Factor (# of cases)	P value (Log-rank)	HR	95% CI Lower	95% CI Upper	P value (PH assumption)	P value (Wald) / BH adj.	HR	95% CI Lower	95% CI Upper	P value (PH assumption)
Gender (female: 40; male: 55)	0.3	1.41	0.78	2.5	0.26	0.07/0.35	1.8	0.94	3.6	0.19
Histo (non SCC: 54; SCC: 41)	0.2	1.43	0.8	2.5	0.56	0.88/0.96	1.05	0.54	2.04	0.92
ECOG (min: 0; median: 0; max: 3)	0.07	1.52	0.969	2.38	0.74	0.13/0.35	1.61	0.875	2.94	0.97
CRT sequence (concomitant: 71; sequential: 24)	0.3	1.38	0.74	2.57	0.41	0.09/0.35	1.92	0.89	4.1	0.34
UICC stage (IIIa: 38; IIIb: 57)	0.01	2.2	1.2	4	0.91	0.08/0.35	1.9	0.92	3.9	0.56
Durvalumab (durvalumab: 63; no durvalumab: 32)	0.007	2.2	1.2	3.8	0.36	0.34/0.51	1.46	0.67	3.2	0.62
Tumor EQD2 (min: 24.8; median: 66; mean: 64.93; max: 100)	0.08	0.98	0.95	1.002	0.18	0.95/0.96	0,9991	0.97	1.028	0.32
LK EQD2 (min: 0; median: 57.29; mean: 50.98; max: 70)	0.8	0.9985	0.98	1.013	0.75	0.88/0.96	0.9985	0.978	1.02	0.71
Tumor GTV (min: 0.8; median: 48.42; mean: 76.79; max: 589.3)	0.006	1.003	1.001	1.005	0.18	0.28/0.49	1.002	0.9987	1.005	0.16
LN GTV (min: 0; median: 25.2; mean: 48.73; max: 473)	0.1	1.004	0.9992	1.009	0.81	0.26/0.48	1.003	0.997	1.009	0.94
Pneumonitis (min: 0; median: 0; max: 4)	0.5	1.1	0.79	1.56	1	0.14/0.35	1.36	0.9	2	0.61
Esophagitis (min: 0; median: 0; max: 3)	0.6	1.08	0.8	1.4	0.8	0.96/0.96	0.9925	0.73	1.344	0.49
elderly (≥ 70 years) patients (N = 65; events = 30)										
	Univariate analysis					Multivariate analysis				
Factor (# of cases)	P value (Log-rank)	HR	95% CI Lower	95% CI Upper	P value (PH assumption)	P value (Wald) / BH adj.	HR	95% CI Lower	95% CI Upper	P value (PH assumption)
Gender (female: 24; male: 41)	0.2	1.6	0.74	3.64	0.41	0.12/0.25	2.08	0.84	5.2	0.76

Histo (non SCC: 37; SCC: 28)	0.9	1.053	0.5	2.2	0.4	0.58/0.64	1.28	0.53	3.1	0.38
ECOG (min: 0; median: 0; max: 2)	0.3	1.4	0.75	2.7	0.15	0.12/0.25	1.8	0.85	3.79	0.02
CRT sequence (concomitant: 48; sequential: 17)	0.2	1.8	0.8	4	0.088	0.11/0.25	2.4	0.83	6.8	0.19
UICC stage (IIIa: 19; IIIb: 46)	0.3	1.62	0.66	3.98	0.86	0.3/0.46	1.68	0.62	4.6	0.67
Durvalumab (durvalumab: 45; no durvalumab: 20)	0.04	2.1	1.01	4.5	0.35	0.04/0.25	2.7	1.05	7	0.67
Tumor EQD2 (min: 32.5; median: 65; mean: 63.04 max 100)	0.4	0.988	0.95	1.02	0.33	0.07/0.25	0.95	0.89	1.003	0. 2
LN EQD2 (min: 0; median: 57.29; mean 49.16; max: 70)	0.2	1.017	0.989	1.05	0.52	0.44/0.58	1.015	0.978	1.05	0.76
Tumor GTV (min: 0.24; median: 49; mean: 92.6; max: 492.9)	0.3	0.998	0.995	1.002	0.27	0.054/0.25	0.995	0.989	1	0.067
LN GTV (min: 0; median: 30; mean: 58.89; max: 285)	0.4	1.002	0.996	1.007	0.64	0.48/0.58	0.997	0.989	1.005	0.39
Pneumonitis (min: 0; median: 0; max: 3)	0.6	0.91	0.6	1.36	0.91	0.89/0.89	0.971	0.63	1.49	0.67
Esophagitis (min: 0; median: 0; max: 2)	0.07	1.42	0.96	2.1	0.25	0.18/0.32	1.41	0.847	2.34	0.55

Supplementary Table S3. Multivariate and univariate analyses of variables in relation to LRC in the < 70 years as well as ≥ 70 years age group. Abbreviations: adj., adjusted. BH., Benjamini-Hochberg and PH., proportional hazards. SCC., Squamous Cell Carcinoma. CRT., chemoradiotherapy. EQD2., Equivalent Dose in 2 Gray (Gy) fractions. LN., lymph nodes. GTV., gross tumor volume.

**Treatment characteristics**										
younger (< 70 years) patients (N = 95, events = 28)										
Factor (# of cases)	Univariate analysis					Multivariate analysis				
	P value (Log-rank)	HR	95% CI		P value (PH assumption)	P value (Wald) / BH adj.	HR	95% CI		P value (PH assumption)
			Lower	Upper				Lower	Upper	
Gender (female: 40; male: 55)	0.8	0.9	0.41	1.94	0.86	0.41/0.999	0.7	0.29	1.6	0.87
Histo (non SCC: 54; SCC: 41)	0.003	3.4	1.45	7.6	0.16	0.004/0.053	4.3	1.4	8	0.1
ECOG (min: 0; median: 0; max: 3)	0.4	1.3	0.689	2.5	0.68	0.98/0.999	1.007	0.45	2.25	0.85
CRT sequence (concomitant: 71; sequential: 24)	0.6	0.75	0.28	1.99	0.55	0.69/0.999	0.78	0.23	2.6	0.3
UICC stage (IIIa: 38; IIIb: 57)	0.1	1.8	0.8	4.2	0.78	0.2/0.79	1.9	0.7	5.1	0.96
Durvalumab (durvalumab: 63; no durvalumab: 32)	0.5	1.35	0.6	3	0.52	0.999/0.999	0.9993	0.3	3.2	0.62
Tumor EQD2 (min: 24.8; median: 66; mean: 64.93; max: 100)	0.4	0.985	0.95	1.02	0.94	0.51/0.999	0.985	0.94	1.03	0.83
LN EQD2 (min: 0; median: 57.29; mean: 50.98; max: 70)	0.9	1.001	0.98	1.02	0.42	0.56/0.999	1.0095	0.978	1.04	0.46
Tumor GTV (min: 0.8; median: 48.42; mean: 76.79; max: 589.3)	0.2	1.002	0.998	1.005	0.24	0.72/0.999	1.0008	0.996	1.005	0.16
LN GTV (min: 0; median: 25.2; mean: 48.73; max: 473)	0.6	1.002	0.99	1.01	0.18	0.83/0.999	1.001	0.99	1.01	0.11
Pneumonitis (min: 0; median: 0; max: 4)	1	0.995	0.65	1.5	0.36	0.76/0.999	1.08	0.65	1.78	0.19
Esophagitis (min: 0; median: 0; max: 3)	0.08	0.69	0.44	1.06	0.15	0.046/0.27	0.62	0.39	0.991	0.17
elderly (≥ 70 years) patients (N = 65; events = 13)										
Factor (# of cases)	Univariate analysis					Multivariate analysis				

	P value (Log-rank)	HR	95% CI		P value (PH assumption)	P value (Wald) / BH adj.	HR	95% CI		P value (PH assumption)
			Lower	Upper				Lower	Upper	
Gender (female: 24; male: 41)	0.2	2.3	0.6	8.4	0.12	0.007/0.04	46	2.79	758	0.15
Histo (non SCC: 37; SCC: 28)	0.3	1.8	0.59	5.3	0.43	0.004/0.044	17.7	2.6	123	0.28
ECOG (min: 0; median: 0; max: 2)	1	0.9994	0.375	2.7	0.28	0.26/0.31	0.38	0.07	2.1	0.09
CRT sequence (concomitant: 48; sequential: 17)	0.01	3.76	1.28	11.5	0.055	0.69/0.69	0.6	0.05	6.8	0.41
UICC stage (IIIa: 19; IIIb: 46)	0,9	0,906	0,278	2,95	0,68	0.097/0.17	0,28	0,06	1,26	0,96
Durvalumab (durvalumab: 45; no durvalumab: 20)	0.5	1.6	0.479	5.1	0.74	0.07/0.17	5.28	0.87	32	0.63
Tumor EQD2 (min: 32.5; median: 65; mean: 63.04 max 100)	0.8	0.993	0.946	1.04	0.46	0.47/0.52	0.94	0.8	1.11	0.38
LN EQD2 (min: 0; median: 57.29; mean 49.16; max: 70)	0.09	1.06	0.99	1.13	0.42	0.0034/0.1	1.26	1.04	1.52	0.95
Tumor GTV (min: 0.24; median: 49; mean: 92.6; max: 492.9)	0.8	0.9993	0.99	1.004	0.8	0.22/0.31	1.007	0.995	1.018	0.63
LN GTV (min: 0; median: 30; mean: 58.89; max: 285)	0.03	1.007	1.001	1.014	0.76	0.01/0.046	1.03	1.006	1.055	0.39
Pneumonitis (min: 0; median: 0; max: 3)	0.7	1.1	0.6	1.93	0.89	0.098/0.17	2.1	0.87	4.98	0.99
Esophagitis (min: 0; median: 0; max: 2)	0.2	1.48	0.8	2.7	0.13	0.24/0.3	0.5	0.18	1.5	0.56

Supplementary Table S4. Multivariate and univariate analyses of variables in relation to LC in the < 70 years as well as ≥ 70 years age group. Abbreviations: adj., adjusted. BH., Benjamini-Hochberg and PH., proportional hazards. SCC., Squamous Cell Carcinoma. CRT., chemoradiotherapy. EQD2., Equivalent Dose in 2 Gray (Gy) fractions. LN., lymph nodes. GTV., gross tumor volume.

**Treatment characteristics**										
younger (< 70 years) patients (N = 95, events = 26)										
Factor (# of cases)	Univariate analysis					Multivariate analysis				
	P value (Log-rank)	HR	95% CI		P value (PH assumption)	P value (Wald) / BH adj.	HR	95% CI		P value (PH assumption)
			Lower	Upper				Lower	Upper	
Gender (female: 40; male: 55)	0.5	0.76	0.34	1.7	0.79	0.09/0.27	0.44	0.17	1.13	0.97
Histo (non SCC: 54; SCC: 41)	<0.001	4.3	1.8	10.4	0.32	<0.001/0.03	4.3	1.7	11.2	0.3
ECOG (min: 0; median: 0; max: 3)	0.2	1.5	0.77	2.84	0.63	0.92/0.93	1.04	0.46	2.4	0.84
CRT sequence (concomitant: 71; sequential: 24)	0.6	0.8	0.29	2.1	0.25	0.92/0.93	0.94	0.28	3.1	0.15
UICC stage (IIIa: 38; IIIb: 57)	0.02	2.8	1.1	7.1	0.85	0.04/0.17	3.3	1.06	10.1	0.5
Durvalumab (durvalumab: 63; no durvalumab: 32)	0.3	1.6	0.7	3.6	0.57	0.62/0.93	1.4	0.39	4.8	0.64
Tumor EQD2 (min: 24.8; median: 66; mean: 64.93; max: 100)	0.4	0.985	0.95	1.02	0.89	0.77/0.93	0.99	0.95	1.04	0.97
LN EQD2 (min: 0; median: 57.29; mean: 50.98; max: 70)	0.8	0.998	0.978	1.02	0.54	0.84/0.93	0.997	0.96	1.03	0.69
Tumor GTV (min: 0.8; median: 48.42; mean: 76.79; max: 589.3)	0.2	1.002	0.998	1.006	0.62	0.66/0.93	0.999	0.995	1.003	0.38
LN GTV (min: 0; median: 25.2; mean: 48.73; max: 473)	0.7	1.002	0.99	1.01	0.17	0.93/0.93	0.9996	0.98	1.009	0.09
Pneumonitis (min: 0; median: 0; max: 4)	0.9	0.98	0.62	1.54	0.35	0.93/0.93	0.974	0.55	1.7	0.147
Esophagitis (min: 0; median: 0; max: 3)	0.06	0.59	0.42	1.03	0.19	0.04/0.17	0.59	0.35	0.98	0.23
elderly (≥ 70 years) patients (N = 64; events = 11)										

Factor (# of cases)	Univariate analysis					Multivariate analysis				
	P value (Log-rank)	HR	95% CI		P value (PH assumption)	P value (Wald) / BH adj.	HR	95% CI		P value (PH assumption)
			Lower	Upper				Lower	Upper	
Gender (female: 24; male: 40)	0.3	1.9	0.5	7.34	0.08	0.01/0.08	21.3	2	219	0.3
Histo (non SCC: 37; SCC: 27)	0.7	1.3	0.38	4.2	0.32	0.02/0.11	11	1.46	86	0.24
ECOG (min: 0; median: 0; max: 2)	1	1.006	0.3	2.9	0.62	0.67/0.81	0.67	0.11	4.2	0.5
CRT sequence (concomitant: 47; sequential: 17)	0.002	5.5	1.7	18.1	0.016	0.79/0.86	1.49	0.08	27.4	0.09
UICC stage (IIIa: 18; IIIb: 46)	0.5	0.69	0.2	2.35	0.63	0.045/0.09	0.19	0.038	0.97	0.9
Durvalumab (durvalumab: 44; no durvalumab: 20)	0.7	1.3	0.35	5.1	0.76	0.24/0.36	3.34	0.4	25	0.93
Tumor EQD2 (min: 32.5; median: 65; mean: 63.09 max 100)	0.9	0.995	0.94	1.05	0.26	0.23/0.36	0.89	0.7	1.08	0.26
LN EQD2 (min: 0; median: 57.29; mean 49.93; max: 70)	0.07	1.09	1.002	1.19	0.065	0.04/0.1	1.25	1.007	1.4	0.48
Tumor GTV (min: 0.24; median: 48.35; mean: 91.72; max: 492.9)	0.7	0.999	0.99	1.005	0.5	0.59/0.79	1.003	0.99	1.02	0.68
LN GTV (min: 0; median: 31.75; mean: 59.81; max: 285)	0.01	1.009	1.002	1.02	0.85	0.029/0.086	1.03	1.002	1.05	0.78
Pneumonitis (min: 0; median: 0; max: 3)	0.5	1.25	0.66	2.35	0.51	0.04/0.09	3.2	1.04	9.64	0.58
Esophagitis (min: 0; median: 0; max: 2)	0.04	1.88	0.98	3.6	0.035	0.91/0.91	0.94	0.32	2.7	0.15
